# *Sporothrix* is neglected among the neglected

**DOI:** 10.1371/journal.ppat.1012898

**Published:** 2025-03-06

**Authors:** Daniel R. Matute, Marcus de Melo Teixeira

**Affiliations:** 1 Biology Department, University of North Carolina at Chapel Hill, Chapel Hill, North Carolina, United States of America; 2 Núcleo de Medicina Tropical, University of Brasília, Brasilia, Brazil; University of Maryland, Baltimore, UNITED STATES OF AMERICA

Fungal diseases are a growing threat to human health. The last 50 years have seen an increase in the prevalence of fungal infections [1,2]. Annually, close to 1 billion people are affected by mycoses [1,3]. Close to 7 million of these infections are life-threatening, and approximately 2 million of them are fatal [3]. One of the fungal diseases that has increased in importance in the last 50 years is sporotrichosis, a mycosis that is caused by members of the genus *Sporothrix*, and that usually manifests as a subcutaneous granulomatous disease [4–9]. Transmission typically occurs transcutaneously after contact with contaminated materials, often during farming or gardening, which can introduce the fungus through skin punctures [6]. The clinical presentation of sporotrichosis varies based on the type of infection and the immune status of the host. The most common forms of sporotrichosis are fixed cutaneous and lymphocutaneous sporotrichosis. In fixed cutaneous sporotrichosis, characterized by initial lesions that resemble small nodules, which may ulcerate over time (sporotrichotic chancres). These lesions are typically painless and remain around the puncture site (fixed). If the lesions spread, they can lead to cutaneous lymphocutaneous sporotrichosis, in which the infection spreads along the lymphatic vessels, resulting in additional nodules and systemic symptoms, or might even develop into disseminated cutaneous sporotrichosis. Extracutaneous forms of sporotrichosis also exist, but they are less common. Ocular sporotrichosis mostly often affects the ocular adnexa, but in some rare instances can also infect intraocular tissues (reviewed in [10]). Pulmonary infection, caused by inhalation of conidia from the air, can be localized or multifocal and is relatively uncommon (~1,000 cases), although these numbers might be artificially low due to underreporting [11]. Finally, osteoarticular infection usually manifests with tenosynovitis, joint effusion, bursitis, and synovial cyst formation [12].

## Sporothrix

Sporotrichosis is caused by a few of the species of the genus *Sporothrix* (Sordariomycetes, Ascomycota), a genus that includes over 100 species [13–15]. The most common etiologic agents of the disease, *Sporothrix schenckii sensu stricto*, *Sporothrix globosa*, and the emerging species *Sporothrix brasiliensis*, were once considered a single species [16,17]. The three species show extensive differentiation and marked phenotypic differences [8,18,19], which may contribute to its greater virulence in animal models. A fourth species in the *schenckii* species complex, *Sporothrix luriei* has been reported to cause rare fixed sporotrichosis in Africa [20] and Italy [21].

The species within the *schenckii* complex were once the only species known to cause disease in humans. Nonetheless, species that are not members of the *schenckii* complex can also be human pathogens. *Ophiostoma* is thought to encompass the presumptive sexual stage of *Sporothrix*, and two species within this genus, *Ophiostoma stenoceras* [22,23] and *Ophiostoma picae* [24], can cause opportunistic sporotrichosis. A seventh species, *Sporothrix humicola*, is known to have caused sporotrichosis in a diabetic cat [25], but whether it can cause disease in humans has not been studied. The phylogenetic relationships between these *Ophiostoma* species, and the pathogenic species of *Sporothrix* remains to be resolved ([26] *cf.* [27]). *Sporothrix chilensis*, *Sporothrix mexicana*, and *Sporothrix pallida*, all within the *pallida* species complex can cause disease in humans but the symptomatology of the disease is different from each other. While *S. mexicana* and *S. pallida* can cause a chronic subcutaneous disease consistent with classical forms of sporotrichosis [28,29], the only known *S. chilensis* clinical isolate associates closely to an environmental sample collected in Chilean soil [30].

**Fig 1:**
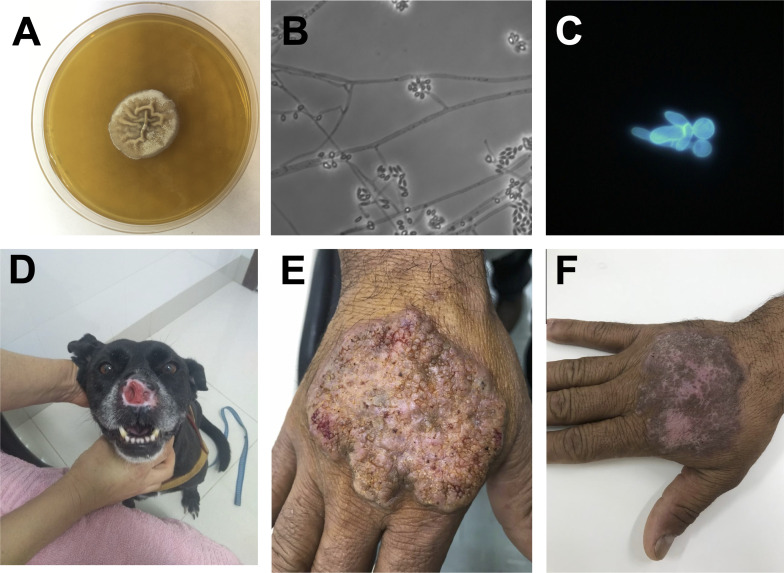
Sporotrichosis and its etiological agent. (**A**) Macromorphology of *Sporothrix brasiliensis* strain A001 mycelial colony after 15 days of growth on mycelial agar, isolated from a cat infected with sporotrichosis. (**B**) Filamentous form of *S. brasiliensis* strain A001 grown on Sabouraud agar by the slide culture method for 15 days. The conidiophores of *S. brasiliensis* are short, branched, and form a “rosette-like” arrangement, producing smooth-walled, one-celled conidia that are oval to round (2–4 µm), clustering at the tips or sides of the conidiophores, supported by septate hyphae. (**C**) Yeast cells of *S. brasiliensis* strain IPEC 16490 grown at 37 °C on BHI medium for 15 days, stained with calcofluor-white, and observed under fluorescence microscopy. (**D**) Lesion on the snout of a dog infected with *S. brasiliensis*, presenting as a raised, ulcerated nodule. (**E**) Classic lymphocutaneous lesion of sporotrichosis. (**F**) Lymphocutaneous lesion of sporotrichosis after treatment with itraconazole. Credits: 1D: João Eudes Filho, 1E, 1F: Dr. Ciro Martins Gomes.

All pathogenic species of *Sporothrix* are thermally dimorphic, and transition between mycelium (the infective form) and yeast (the pathogenic form) is cued on environmental temperature (Fig 1). The transition can be affected by pH and carbon source [31]. Whether thermoregulation of dimorphism is a genus trait or, on the contrary, is limited to the pathogenic species remains unexplored. A systematic assessment to determine whether there are other *Sporothrix* species that are human pathogens, even if opportunistic, is urgent (Box 1).

## Ecology and geographic range of *Sporothrix*

For over 100 years, sporotrichosis was recognized as a sapronotic disease, primarily transmitted through traumatic inoculation of fungal fragments from plant-derived material [6]. This classical route of transmission is widespread across a large swath of the geographic range of the three main species of pathogenic *Sporothrix*. Nonetheless, the disease has been recently recognized to not only be saprobiotic but also zoonotic. Several animals can serve as reservoirs of *Sporothrix*, which have the ability to infect a variety of mammals in addition to humans, including mice, dogs, and especially cats [32]. The geographic ranges of the pathogenic species remain speculative and increased sampling from environmental samples is sorely needed to understand the epidemiology and sources of infection by *Sporothrix* [33,34].

The true magnitude of sporotrichosis as a public health issue remains unknown. Since sporotrichosis is not a mandatory reportable disease, the prevalence and burden of the disease remain mostly unmeasured. Currently, sporotrichosis is known to be common and widespread across multiple countries, but is especially frequent in Latin America, Southern Africa, and Asia [5,8,35]. Hyperendemic regions in South America show an incidence that might range from 48 to 98 cases per 100,000 persons [5], but this number is likely to be an underestimation (Box 1). In each of these locations, the disease has particular epidemiological aspects that need to be taken into account. For example, in South Africa the disease is common among gold mine workers [33,36] which has been attributed to the humid conditions of the mines and the presence of mining timber [37].

The epidemiological profile of sporotrichosis has shifted dramatically over the past 30 years. The case of *S. brasiliensis* is of note. Since the 1990s, local outbreaks caused by *S. brasiliensis* have been detected in Rio de Janeiro, Brazil, resulting from cat bites and scratches [38]. Cat-transmitted sporotrichosis by *S. brasiliensis* is found throughout South America [39], and more recently in the United Kingdom [25,40]. Future studies need to address whether sporotrichosis cases, caused by all of the different species of *Sporothrix* are on the rise together, or if a particular species shows more cause for concern than the others (Box 1).

The increase in the relative importance of sporotrichosis among mycoses can be attributed to a number of factors. First, the number of cases might reveal an expansion of the geographic range, increased ability to cause infection, or increased ecological opportunity. A second possibility is that the increased number of cases stems from more systematic vigilance for the disease. These are questions that can, and should, be addressed in the near future.

## Virulence, antifungal resistance, and other health-related traits

Most of the knowledge regarding virulence in *Sporothrix* has been extrapolated from other fungal pathogens. The production of melanin, adhesins, and ergosterol peroxide are traits that have been hypothesized to be involved in virulence in *Sporothrix* [4]. Similar to other dimorphic fungi, thermotolerance has also been implicated in virulence. Yet, no direct tests exist for any of these hypotheses. Phenotypic assays have revealed the existence of variation in traits potentially involved in virulence such as melanin, or urease production in both *S. schenckii* and *S. brasiliensis* [41]. Mouse experiments showed that seven of the eight pathogen species persisted in the host, to different degrees [42]. *S. brasiliensis* is the most virulent species in terms of mortality, tissue burden, and tissue damage, followed by *S. schenckii*. Other species like, *S. mexicana, S. chilensis*, and *O. stenoceras* show low virulence in mouse models [42,43].

The identity of virulence factors in *Sporothrix* remains speculative. Comparative genomics has revealed that virulence factors from other fungi are present in the *S. schenckii* genome [44], but no causal tests have tested their role in pathogenesis in *Sporothrix*. The recent implementation of CRISPR in *S. schenckii* and *S. brasiliensis* [45] should open the door to genetically testing allele involvement in virulence, and facilitate a comparative study of the genetic architecture of different virulence genes in the *Sporothrix* genus.

Antifungal resistance in *Sporothrix* has become a growing public health concern, especially for the three species within the *schenckii* species complex. Recent studies have identified strains resistant to itraconazole, the first-line treatment, as well as to amphotericin B, a last-resort option, in isolates from *S. schenckii*, *S. globosa*, and *S. brasiliensis* [16,46]. The molecular basis of this resistance is yet to be genetically dissected.

## Challenges and future directions

As an emergent pathogen, *Sporothrix* represents a public health challenge. Even though *Sporothrix* was not included in the World Health Organization (WHO) fungal priority list [47], the preponderance of increasing caseloads, novel antifungal resistance, and geographic range expansion suggests that *Sporothrix* merits serious attention. While the number of cases of sporotrichosis and the scholarly output in the pathogen has grown over the last decade [48].

Questions about the evolutionary biology of *Sporothrix* remain largely unexplored. The nature of the adaptation of *Sporothrix* to new environments, and forecasts of its potential responses to global warming are pressing questions (Box 1). Mycoses outbreaks often occur after natural disasters strike [49]. Indeed, sporotrichosis outbreaks have been associated with extreme weather events, such as severe flooding [5,50]. Such flooding events are expected to increase in frequency and severity as the planet warms [49]. Population genetics provides the tools to study the tempo and mode of environmental adaptation within each species of *Sporothrix*, offering the opportunity to address these and similar questions.

A similar paucity also exists in understanding the processes that drive divergence above species-level. Relationships between species have only been studied using multilocus sequence typing [27,30] or microsatellites [51], which are good exploratory tools but are limited in their scope. Proper assessments of phylogenetic relationships, extent of divergence, and estimates of admixture among lineages using whole genome data are all necessary to determine the relative importance of different evolutionary processes in *Sporothrix*. Whether other species of *Sporothrix*, besides the *schenckii* complex and the minor pathogens mentioned above, are pathogenic to mammals remains unknown [34]. A second potential line of research is to quantify the range of animal reservoirs for different species of *Sporothrix* and evaluate the extent of host-pathogen range overlap and coevolution. Genomics combined with systematic sampling has the power to address these questions.

We hypothesize the burden of sporotrichosis will grow in the future for at least three reasons. First, the zoonotic transmission potential of *Sporothrix* species poses a global threat as evidenced by the cat-induced transmission across the world [19,25,40]. The patterns of pet global movement and the increase of the number of potential reservoirs (e.g., pet and feral cats) makes this a concern. Second, as global warming accelerates, *Sporothrix* is one of the fungal pathogens that might expand its host and geographic ranges [50,52]. Finally, antifungal resistance seems to be widespread across several species of *Sporothrix* [53]. Understanding the life cycle and the evolutionary drivers of the pathogen before it becomes an epidemic problem will certainly have an important and positive impact on future public health.

Box 1: **Unsolved questions in *Sporothrix* and sporotrichosis**

1. What is the true disease burden, prevalence, and incidence of sporotrichosis?
2. Do sporotrichosis outbreaks occur because of increased human–animal reservoir contract, novel pathogen variants, or a combination of both?
3. Can genomics and metagenomics be used to collect pathogenic species in their natural habitat?
4. Are there host populations that are more vulnerable to sporotrichosis?
5. What is the geographic range and ecological reservoirs of each species of *Sporothrix*?
6. How many species of *Sporothrix* are pathogenic?
7. What is the genealogical history of the different portions of the *Sporothrix* genome?
8. Is multilocus sequence typing predictive of the phylogenetic relationships between *Sporothrix* and *Ophiostoma* species?
9. Is there opportunity for species interbreeding and hybrid lineages in *Sporothrix*?
10. Does virulence in *Sporothrix* have a single common origin or has it emerged multiple times in the evolutionary history of the genus?
11. What is the adaptation potential of *Sporothrix* to a changing planet? Is adaptation limited by new mutations or does it take place through standing variation?
12. Is there host-pathogen coevolution between animal reservoirs and the different species of *Sporothrix*?
13. What is the identity of virulence factors and alleles involved in antifungal resistance?
14. Is there a need for a vaccine against *Sporothrix*? If so, what is the best strategy and candidates?
